# Intraspinal Transplantation of the Adipose Tissue-Derived Regenerative Cells in Amyotrophic Lateral Sclerosis in Accordance with the Current Experts' Recommendations: Choosing Optimal Monitoring Tools

**DOI:** 10.1155/2018/4392017

**Published:** 2018-08-12

**Authors:** Magdalena Kuzma-Kozakiewicz, Andrzej Marchel, Anna Kaminska, Malgorzata Gawel, Jan Sznajder, Anna Figiel-Dabrowska, Arkadiusz Nowak, Edyta Maj, Natalia Ewa Krzesniak, Bartlomiej H. Noszczyk, Krystyna Domanska-Janik, Anna Sarnowska

**Affiliations:** ^1^Department of Neurology, Medical University of Warsaw, Zwirki i Wigury 61 Str, 02-091 Warsaw, Poland; ^2^Neurodegenerative Diseases Research Group, Medical University of Warsaw, Zwirki i Wigury 61 Str, 02-091 Warsaw, Poland; ^3^Department of Neurosurgery, Medical University of Warsaw, Zwirki i Wigury 61 Str, 02-091 Warsaw, Poland; ^4^Department of Rehabilitation, Józef Piłsudski University of Physical Education in Warsaw, Marymoncka Str. 34, 00-968 Warsaw, Poland; ^5^Mossakowski Medical Research Centre, Polish Academy of Sciences, 5 Pawinskiego Str, 02-106 Warsaw, Poland; ^6^2nd Department of Clinical Radiology, Medical University of Warsaw, Zwirki i Wigury 61 Str, 02-091 Warsaw, Poland; ^7^Department of Plastic and Reconstructive Surgery, Centre of Postgraduate Medical Education, Prof. W. Orlowski Memorial Hospital, Czerniakowska 231, 00-416 Warsaw, Poland

## Abstract

Stem cells (SCs) may constitute a perspective alternative to pharmacological treatment in neurodegenerative diseases. Although the safety of SC transplantation has been widely shown, their clinical efficiency in amyotrophic lateral sclerosis (ALS) is still to be proved. It is not only due to a limited number of studies, small treatment groups, and fast but nonlinear disease progression but also due to lack of objective methods able to show subtle clinical changes. Preliminary guidelines for cell therapy have recently been proposed by a group of ALS experts. They combine clinical, neurophysiological, and functional assessment together with monitoring of the cytokine level. Here, we describe a pilot study on transplantation of autologous adipose-derived regenerative cells (ADRC) into the spinal cord of the patients with ALS and monitoring of the results in accordance with the current recommendations. To show early and/or subtle changes within the muscles of interest, a wide range of clinical and functional tests were used and compared in order to choose the most sensitive and optimal set. Additionally, an analysis of transplanted ADRC was provided to develop standards ensuring the derivation and verification of adequate quality of transplanted cells and to correlate ADRC properties with clinical outcome.

## 1. Introduction

Amyotrophic lateral sclerosis is a fatal neurodegenerative disease characterized by a progressive loss of motor neurons in the central and peripheral nervous system. Starting insidiously with an impairment of fine hand movements, foot drop, or slurred speech, in 2 to 5 years it leads to quadriplegia, anarthria, aphagia, and respiratory insufficiency [1]. There is no causative treatment. The first available drug, riluzole, prolongs survival by the average of 3 months, while edaravone, recently registered in Japan and US, relents disease progression in a subgroup of patients without respiratory involvement [2, 3]. Patients with mutations in the *SOD1* gene might in future profit from oligonucleotide strategy, but the clinical trials are still ongoing [4, 5]. Stem cells (SCs) may therefore constitute a perspective alternative to pharmacological treatment in ALS. Although the safety of SC transplantation has been widely shown [6–8], their efficiency in ALS is still unproven. It is not only due to a limited number of studies, high variability of transplanted cell types, small treatment groups, and fast but nonlinear disease progression but also the lack of objective methods able to prove subtle clinical changes.

The usual monitoring strategies used in clinical trials involve the combination of muscle strength assessment: MRC (Medical Research Council), grip test, functional status: ALSFRS R (amyotrophic lateral sclerosis functional rating scale-revised), Norris scale, and respiratory functions: FVC and SNIP. In a recent statement, the representatives of International Workshop on Progress in Stem Cells Research for ALS/MND recommend the use of ALSFRS R, FVC, electrophysiological quantitative motor unit assessment, and cytokine level [9].

Here, we describe a pilot study on transplantation of autologous adipose-derived regenerative cells into the spinal cord of the patients with ALS and monitoring of the results in accordance with the current recommendations. In a limited number of patients, we aimed to use a wide range of clinical and functional tests in order to identify measures able to show early and/or subtle changes within the muscles of interest.

Our choice of the material for transplantation was dictated by a relative safety, nontumorigenic history, and availability of mesenchymal cells [10]. Embryonic stem cells, despite their high ability to differentiate toward neuronal cells, can give rise to tumors. In turn, a very good and quite safe material, such as fetal cells, is still ethically controversial and difficult to obtain for wider therapy. Recent papers present promising findings of preclinical experiments with adipose-derived regenerative cell (ADRC) transplantation. The ADRCs constitute a freshly isolated, heterogeneous population, characterized and described also by our group [10], not cultivated in vitro (in contrast to ADSC) to enhance or keep their high immunomodulatory properties [11]. These cells may be isolated under a moderate level of invasiveness and with comparatively high efficiency per number of cells. Authors demonstrate their remarkably strong adjuvant properties that are thought to be responsible for the positive results in regenerative medicine [12]. Scientific data suggest that ADRCs improve blood flow, modulate the inflammatory response, and protect tissues from dying. Furthermore, compared with the bone marrow stromal cells, ADRCs show greater plasticity, longer survival times, and higher vasculogenetic potential. An ADRC can be induced to differentiate, not only into adipocytes, bone, and chondrocytes in line with basic MSC properties but also under defined conditions into neuroectodermal cell lineages [13]. However, the protective properties of ADRCs depend strictly on their stage-related differentiation. Our data gathered in preclinical and clinical experimentation indicate that freshly isolated, undifferentiated, and highly proliferating MSC have the highest adjuvant properties [11]. With time of culture, cells differentiate and their protective effect declines in parallel with changes in their paracrine/adjuvant capabilities [14].

To correlate properties of freshly isolated ADRC with clinical outcome, each ADRC fraction obtained and ready for transplantation was divided into portions assigned to transplantation and basic research.

## 2. Material and Methods

### 2.1. Study Design

The study was registered at http://ClinicalTrials.gov (NCT03296501). It was designed as a nonrandomized, prospective, single-center, open-label study, with no placebo control, to assess safety and efficacy of ADRC transplantation into the individuals with ALS. After a minimum of 3 months of clinical, functional, and electrophysiological monitoring, the patients underwent ADRC injection followed by 2 subsequent intrathecal infusions. Safety, adverse events, and efficacy were confirmed by clinical, electrophysiological, neuroimaging, and pulmonary function assessment together with functional and objective motor assessment. The quality of life (QoL) and depression were monitored throughout the study.

Both the study protocol and the informed consent procedure were approved by the Bioethics Committee of the Medical University of Warsaw. Prior to enrollment to the study, each participant was given detailed “Information for the study participant” and signed an informed consent in 2 copies, one for each party. The procedure was repeated before each surgical intervention.

### 2.2. Patients

Patients with ALS were diagnosed and followed at the Department of Neurology, Medical University of Warsaw. The qualification procedure included three steps. The first step was a register-based-approach. The patients' clinical data was taken from the Warsaw ALS/MND registry, which collects demographic and clinical information of all ALS/MND patients diagnosed and followed at the Department of Neurology, Medical University of Warsaw, since 2003 (*n* = 780). By using inclusion and exclusion criteria as search filters (Table 1), a group of potentially eligible patients was identified. The second step involved updated clinical assessment. In case of positive verification, the patients signed an informed consent (third step) and were included into the study.

### 2.3. Surgical Procedures

#### 2.3.1. Liposuction

The fat tissue was harvested from the abdomen or inner thighs after infiltration with Klein solution (based on lactated Ringers solution with lidocaine and epinephrine) under local anesthesia. In order to preserve regenerative properties of cells, fat collection was performed in low pressure (250 kPa), using syringe liposuction. We obtained approximately 250 ml of lipoaspirate during each procedure using 3 mm harvesting cannulas [15–17].

#### 2.3.2. Lumbar Laminectomy

The patient was placed in the prone position, and the operative field was aseptically prepared. Fluoroscopy was used to obtain positive confirmation of the appropriate levels for thoracolumbar (T10/11) exposure. A standard midline posterior approach was used with completion of a Th10–Th11 laminectomy. A Surgicel® was used as a hemostatic aid when necessary. A 4–5 cm incision was made through the dura, exposing the spinal cord. Following the dura opening, a microinjection platform was fixed to the operating table. The platform itself consists of 2 rigid bars spaced to provide easy visualization of the dura and the spinal cord. Originally designed gondola was positioned along these rods. It was equipped with universal joints that allowed for correction of the sagittal and coronal angles. The device also allowed for adjustment in the mediolateral and rostrocaudal direction. The dura was reflected away from the pial layer and secured to the operative drape. The dorsal root entry zone was identified, and the pial surface was opened with a microscalpel under operating microscope magnification. The spinal cord was penetrated on an orthogonal trajectory to the cord surface at a point 1 mm medial to the dorsal root entry zone. Patients received four bilateral (8 total) injections performed at 4 mm intervals along the rostrocaudal axis at Th10–Th11. For intraspinal transplantation, the patient received 8 × 10 *μ*l at the concentration of 2000,000/10 *μ*l which resulted in the total cell number of 16,000 cells/80 *μ*l. The injection rate was programmed to 10 *μ*l/min, using a precalibrated microinjector pump. Doses were administered through the pump connected to a syringe and needle assembly. The cell suspension was infused at an average depth of 4 mm from contact at the pial surface after an initial needle introduction to an average depth of 5 mm. Depth coordinates were individualized based on preoperative MRI results and ranged from 3 to 5 mm. By passing to a depth of 5 mm and then pulling back 1 mm, a small reservoir was created to reduce reflux. During the injection process, the mechanical ventilation of the patient was kept shallow in order to reduce ventilation-associated cord excursion. Following infusion, the needle was left in place for 1 extra minute to minimize the potential for cell suspension reflux along the needle track. Following watertight dural closure, muscular and fascial layers were closed. Finally, skin closure was completed. Somatosensory evoked potentials (SSEPs) were monitored throughout the procedure.

#### 2.3.3. Intrathecal Stem Cell Injection

Intrathecal stem cell injections were performed with the use of a lumbar drainage system. Lumbar drain was placed at the level of the L3 or L4 vertebrae, so that the introducing needle entered below the level at which the spinal cord ended. An average of 15 cm of drain was introduced into the subarachnoid space of the vertebral canal thus positioning its end at the level of the Th12 vertebrae. The tubing was then secured onto the skin with a stitch and covered with a clear sterile dressing. A total cell suspension volume of 4 ml (approx. 14,000,000 cells/ml, 56,000,000 cells/4 ml in total) was injected over 2 minutes followed by saline injection (1 ml over 30 sec). The drain was left in place for 24 hours to reduce suspension reflux through the punctured dura.

### 2.4. Patients' Evaluation

**Figure 1:**
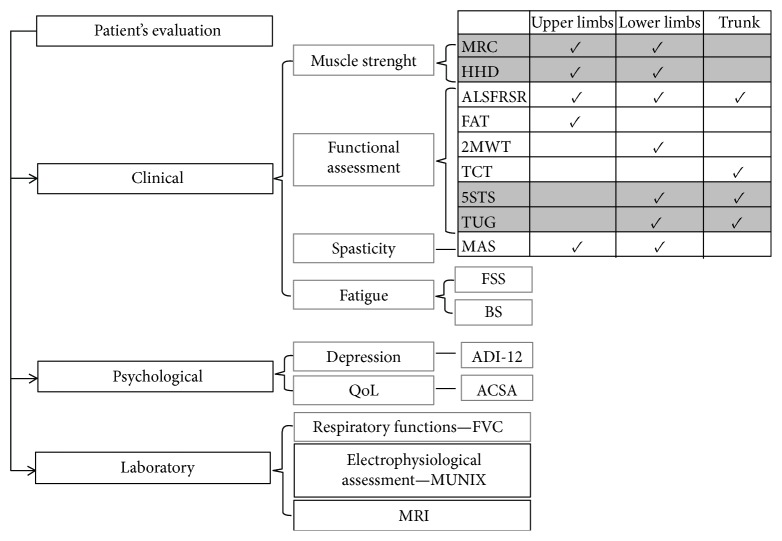
Patient's assessment paradigm. The diagram presents the applied electrophysiological methods, psychological assessment, and functional tests, along with their assignment to specific functions.

The patients' evaluation was divided into three sections: clinical assessment, psychological evaluation, and laboratory tests (Figure 1). The functional assessment was performed by the same physical therapist specialized in neuromuscular diseases. The timetable of the assessments is presented in Table 2. The assessments performed at the visits that involved intervention (either intraspinal or intrathecal ADRC transplantation) were performed 3–7 days prior to a respective intervention.

### 2.5. Muscle Strength

#### 2.5.1. Manual Muscle Test

The manual muscle test was performed according to the Medical Research Council (MRC) [18]. Every assessed muscle group was rated from 0 (no visible contraction) to 5 (normal).

#### 2.5.2. Handheld Dynamometer

(HHD)—MicroFET 2 was used to assess arm flexors, elbow flexors, wrist extensors, finger extensors, abductors of digitus secundus and hip flexors, knee extensors, and ankle dorsiflexors. The result was an average score of three attempts in each muscle group [19].

### 2.6. Functional Assessment

#### 2.6.1. Amyotrophic Lateral Sclerosis Functional Rating Scale-Revised (ALSFRS R)

ALSFRS R contains 12 functional items concerning bulbar function, upper limb, lower limb, trunk, and respiratory functions. Each item is scored from 0 (unable to) to 4 (normal) [20]. The scale was independently applied by a neurologist specializing in ALS and a physiotherapist—interrater differences were discussed prior to final scoring.

#### 2.6.2. Frenchay Arm Test (FAT)

The Frenchay Arm Test was used to assess the function of the upper limbs. It consists of five simple tasks: drawing a line with a ruler, picking up a pin, picking up a glass of water and drinking some, fixing a clothespin to the stick and removing it, and combing hair with scores 0 (unable to) to 1 (able to perform) [21]. It was performed in both upper limbs, and the average score was used in further analysis. In the original version, the affected hand was assessed.

#### 2.6.3. Trunk Control Test (TCT)

The trunk control test was administrated to evaluate functions of the muscles of the trunk [22]. It involves completion of four tasks: rolling to a weak side, rolling to a strong side, balancing in a sitting position, and sitting up from lying down. Each task is scored from 0 (unable to) to 12 (able with help or in abnormal style) to 25 (fully able). The result was expressed in a sum of item scores.

#### 2.6.4. Two-Minute Walk Test (2MWT)

The two-minute walk test was used to evaluate the function of lower limbs and trunk muscles. The results were expressed in distance (meters); the patient was able to cover in 2 minutes [23]. The level of fatigue was additionally estimated before and after the test by rate of perceived exertion (Borg scale (BS)) [24].

#### 2.6.5. Five Times Sit to Stand Test (5STS)

5STS was also used to measure functional muscle strength of lower extremities and trunk [25]. A patient's task was to rise from a chair five times as fast as possible with arms crossed on the chest. The results were expressed in seconds as a mean of 3 independent attempts.

#### 2.6.6. Timed Up and Go (TUG)

TUG similarly assessed the functional use of the lower extremity and trunk when a patient was asked to rise from an armchair, walk 3 meters, turn, walk back, and sit down again [26, 27]. The results were expressed in seconds as a mean of 3 independent attempts.

### 2.7. Spasticity

#### 2.7.1. Modified Ashworth Scale (MAS)

MAS assesses muscle tone in the upper and lower limbs [28, 29]. It is scored from 0 (normal muscle tone) to 4 (affected part rigid in flexion or extension of extremity). The results were expressed as a mean of the results obtained in all limbs.

### 2.8. Fatigue

#### 2.8.1. Fatigue Severity Scale (FSS)

FSS consists of 9 points scored from 1 (definitely disagree) to 7 (definitely agree). A result of >36 is considered positive [30].

### 2.9. Depression

#### 2.9.1. ALS Depression Inventory (ADI-12)

ADI-12 is a self-reported questionnaire developed specifically to screen for depression in ALS [31], describing mood, anhedonia, and energy, without referring to motor-related symptoms. It consists of 12 statements concerning the past two weeks, scoring from 12 (best possible) to 48 (worst possible). Scores between 23 and 29 indicate mild depression and 30 as severe depression.

### 2.10. Quality of Life

### 2.11. Anamnestic Comparative Self-Assessment (ACSA)

ACSA assesses global QoL compared to the worse and the best time of a lifetime scoring from −5 (as bad as possible) to +5 (as good as possible) [32].

### 2.12. Electrophysiological Evaluation

#### 2.12.1. Motor Unit Number Index (MUNIX)

The MUNIX was performed according to the protocol developed by Neuwirth and Weber [33]. Six muscles were examined on both sides: abductor pollicis brevis, abductor digiti minimi, biceps brachii, tibialis anterior, extensor digitorum brevis, and abductor hallucis muscles after supramaximal distal stimulation of the median, ulnar, musculocutaneous, peroneal, and tibial nerves, respectively. The results (CMAP amplitude in mV) and motor unit number index were analyzed by a certified neurophysiologist. The results were expressed as the sum of results from all extremities (total MUNIX), upper and lower limbs (MUNIX upper limbs and MUNIX lower limbs).

### 2.13. Cell Isolation and Analysis

#### 2.13.1. Adipose-Derived Regenerative Cell (ADRC) Isolation

Preclinical characterisation of ADRC cellular composition, rate of proliferation (PDT), genetic stability under 5% oxygen, and maintenance of stemness properties together with commitment to cell differentiation toward mesodermal direction were described in our previous publication [10].

Under operating room conditions, immediately after collection, the lipoaspirate was processed in the CellCelution 800 System (Cytori Therapeutics Inc., San Diego, CA). It was washed to remove free blood and lipids and digested with Celase 800 (Cytori Therapeutics Inc.) enzyme to release the stromal vascular fraction. After a series of centrifugation steps and passing through a system of sieves with various pores, the stromal fraction was concentrated, and 5 ml of ADRC suspended in Ringer solution was prepared for transplantation: 4 ml was intended for patient's treatment, while 1 ml of remnant stromal vascular fraction was used for *in vitro* analysis.

#### 2.13.2. CFU-F Assay

The ADRC at 0 passage was seeded on 6-well plates at a density 100 cells/well. The seeded cells were cultured in standard culture condition for 10 days. Then, the cells were fixed with 4% PFA for 15 minutes and stained with 0.5% toluidine blue for 20 minutes. The 1% stock solution of dye was prepared in 70% ethanol, and distilled water was added in proportion 1 : 1 to obtain the final 0.5%-mixture concentration. After incubation, the stain was directly washed with distilled water. The ADRC-stained colonies containing 50 or more cells were counted, and CFU-F frequency (number of colonies per number seeded cells) was calculated.

#### 2.13.3. Determination of Chemokine and Cytokine Profile

**Figure 2:**
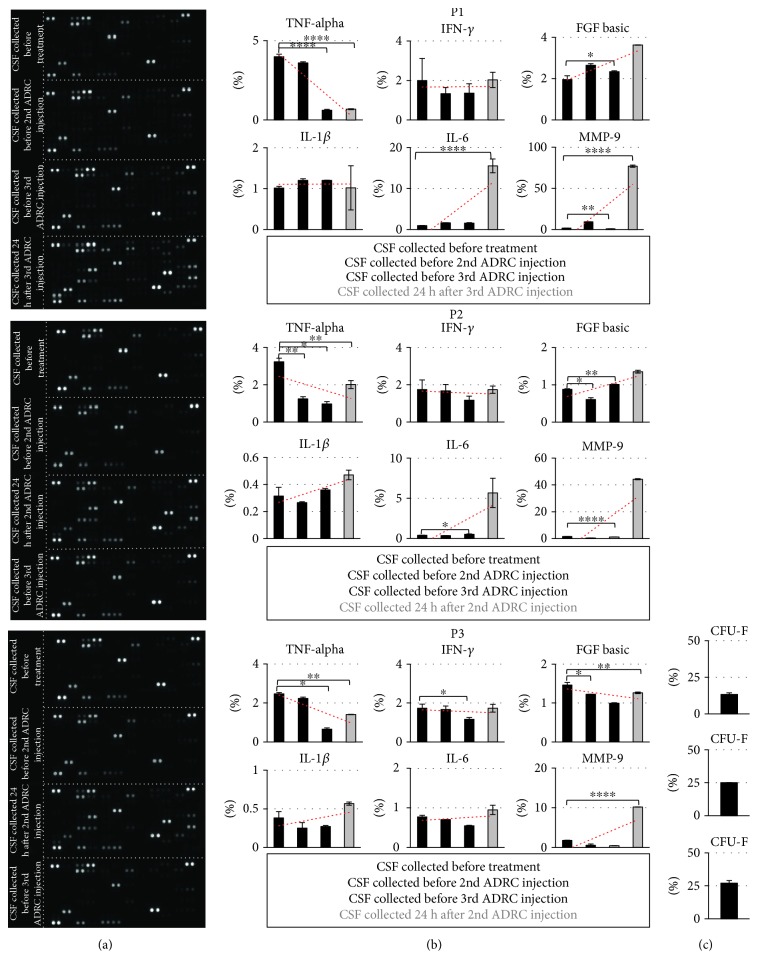
Cytokine and chemokine level in CSF after ADRC application. (a) Membrane-based antibody arrays for the parallel determination of the relative levels of human cytokines and chemokines from CSF of ALS patients (resp. P1, P2, and P3). (b) Comparison of TNF*α*, IFN-*γ*, IL-1*β*, IL-6, bFGF, and MMP-9 level after MSC transplantation ( black graph bars—3 months after ADRC application, grey bars—24 hours after cell application). (c) Quantification of CFU frequency. ADRCs obtained after enzymatic isolation were analyzed to evaluate the stem cell/clonogenic population among a heterogeneous stromal cell population.

The analysis of 102 chemokines or cytokines was performed on CSF samples using the Proteome Profiler Human XL Cytokine Array Kit (R&D Systems) following manufacturers' protocol (Table 3). The kit consists of 4 membranes, which were incubated overnight with 300 *μ*l of every sample and a binding buffer. The membranes were washed and incubated with the detection cocktail, with streptavidin-conjugated horseradish peroxidase (R&D Systems) for 30 min and finally incubation with the chemiluminescence reagent. The spots were visualized by Fusion FX6 (Vilber Lourmat) with EvolutionCapt FX6. The signal readings were proportional to the amount of the bound analyte. The results were analyzed by Zen Software (Zeiss) based on densitometric analysis (Figure 2). The graph bars present means from two spots captured with analyzed antibody according to the following formula: mean density of analyzed spots/mean density of control spots × 100%−mean density of background.

### 2.14. Statistical Analysis

Statistical analysis of the raw data was conducted using the GraphPad Prism version 5 software (La Jolla, CA, USA). The mean ± SEM was calculated for all samples, and significance was determined using one-way ANOVA followed by adequate post hoc test. For data of MUNIX and HHD evaluation, the Pearson coefficient was calculated. The values were considered as significant with a value of *p* < 0.05 (^∗^*p* < 0.05, ^∗∗^*p* < 0.01, ^∗^*p* < 0.001, and ^∗∗∗∗^*p* < 0.0001).

## 3. Results

### 3.1. Clinical Outcome

**Figure 3:**
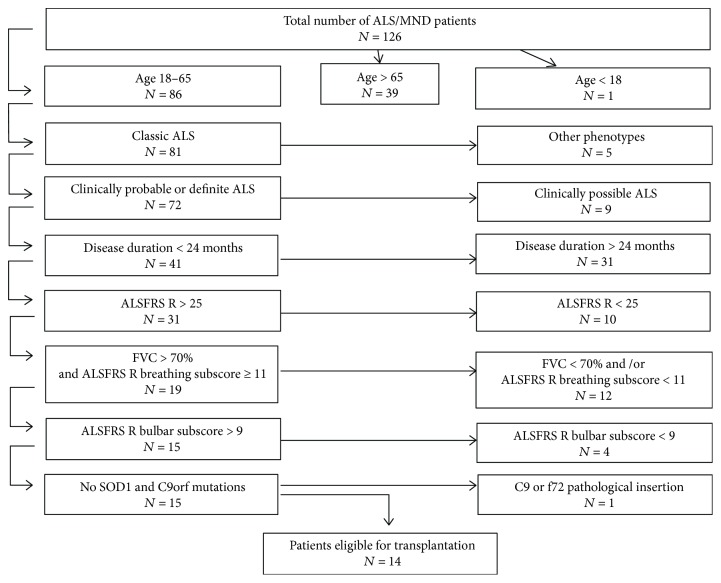
The qualification scheme of patients according to the proposal during the International Workshop on Progress in Stem Cells Research for ALS/MND in 2016.

One hundred and twenty six patients were diagnosed with MND between January 2015 and November 2017 at the Department of Neurology and/or at the University MND outpatient clinic. Of these, 86 patients were at the age 18–65 (Figure 3). From the remaining group, 5 patients had clinical phenotypes other than classic ALS: progressive muscle atrophy (*n* = 2), flail arm or flail leg syndrome (*n* = 2), or primary lateral sclerosis (*n* = 1). Nine patients presented clinically possible ALS according to the El Escorial criteria [34], 31 had a disease duration longer than 24 months, and 10 patients were at the advanced disease stage (ALSFRS R < 25). From the remaining group of 31 patients, 12 had respiratory insufficiency and 4 a marked bulbar involvement. One patient was excluded from the study due to a mutation in the C9orf72 gene. All in all, only 11% of patients (*n* = 14) fulfilled the inclusion criteria. The updated clinical assessment disqualified 9 patients as no longer eligible for the study due to either gastrostomy, respiratory insufficiency, or loss of walking capacity. One patient decided to rebuff the transplantation.

The transplantation of ADRC into the spinal cord at the level of Th10–11 was performed in four patients (3.2%, 3 males/1 female, mean age 37.2, site of onset—upper limbs (4/4), mean diagnosis delay 14.7 months, and mean disease duration 19.0 months). One patient underwent additionally intraspinal transplantation into the cervical part. The results of this application will be published separately. In all cases, intraspinal injection was followed by two intrathecal cell infusions performed in subsequent 3-month intervals. The patients were followed every 3 months.

There was no death related to the transplantation procedure. One patient died 29 months after the first transplantation, two days after gastrostomy insertion. The autopsy was not performed. The adverse events (AEs) included abdominal pain (2/4 patients, severity 1–3/10) in the area of liposuction, which resolved within 24–36 hours after the intervention, and a persistent superficial sensation impairment in the close surrounding of the site of cannula insertion. In case of the intraspinal transplantation, all patients experienced pain at the site of laminectomy (severity 5–6/10), which required analgesic therapy for 7–10 days. One patient reported intubation-induced speech difficulties, which resolved within 10 days without treatment. Following intrathecal infusion, one patient experienced headache without signs of meningeal irritation.

**Figure 4:**
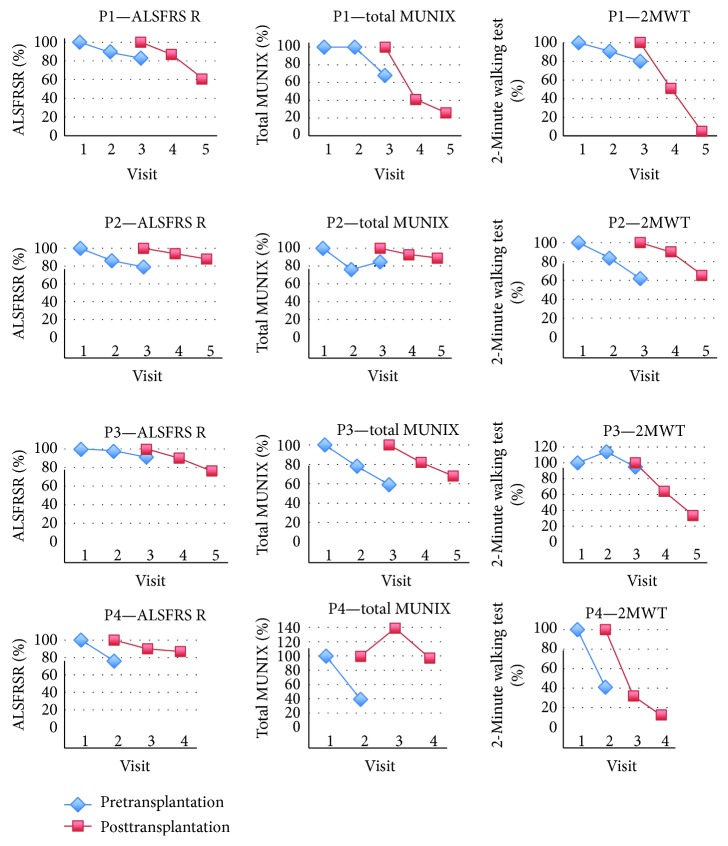
Functional tests. According to Figure 1, functional tests evaluating muscle efficiency in the upper (FAT) and lower limbs (2MWT, TUG, and 5STS) and trunk (TCT) were performed in several time points before, in the course of, and after ADRC therapy. The stability or decrease in these functions was compared with the trajectory of the ALSFRS scale. Impairment in all functional tests was observed faster than decrease in ALSFRS. Although 5STS and TUG showed good correlation with other functional parameters, their longitudinal use was limited in patients who were no longer able to stand up without the help of their arms/hands (on average 6–9 months after the first transplantation; P3 and P4).

Two patients reported reduction of previously increased muscle tone immediately after intraspinal ADRC infusion. Although the study timetable did not include 2MWT and BS evaluation directly after the intervention, in case of patient P2, the tests were additionally carried following the patient-reported walking improvement. The outcome of the 2WMT improved from 100.5 m (fatigability of 8 at BS) prior to transplantation to 128.0 m (with markedly reduced fatigability—BS of 5) seven days after the procedure. There was a slight reduction in the progression of walking impairment: from a 30% reduction of the 2MWT in the 3 months before the operation to a 10% drop in the following observation period (Figure 4). Patient P4 reported improved walking, arm movement, and speech from the second week after transplantation. An objectification in the direct postoperative period was not possible due to the pain in the lumbar region. At three months after transplantation, there was no apparent reduction of a walking capacity slope. According to both patients, the effects persisted for 4 weeks and did not occur after any of the intrathecal infusions.

Both pre- and posttransplantation monitoring showed a physical, functional, and respiratory decline in all studied patients (Figure 4, Table 4).

**Figure 5:**
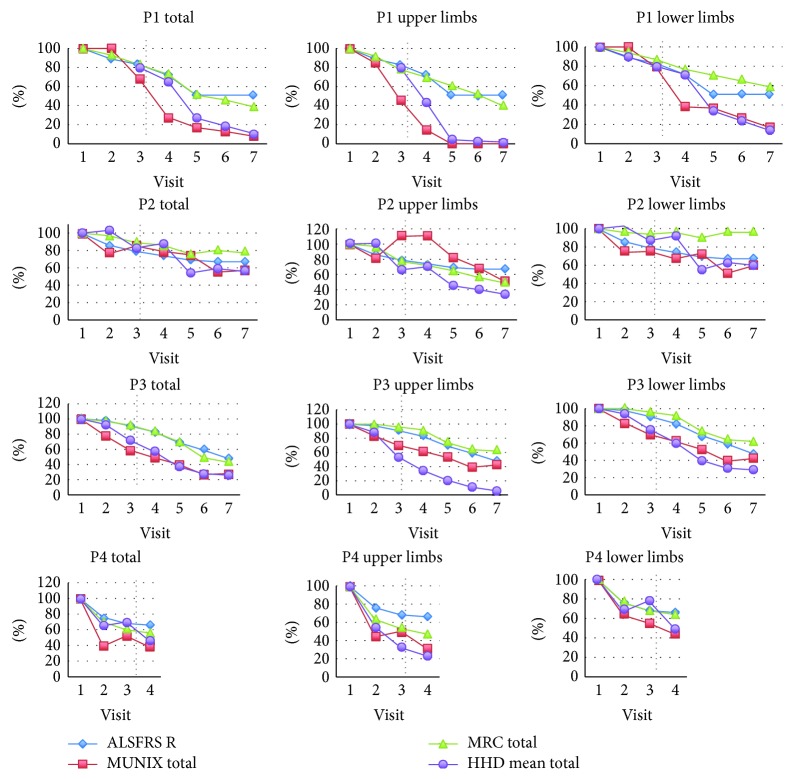
Muscle strength and electrophysiological assessment. The detailed clinical evaluation performed within one week of the neurosurgery procedure and repeated every 3 months throughout the project was complemented with ALS-FRS-R, MRC, MUNIX, and HHD evaluation. ALSFRS R and MRC shared a similar deterioration pattern. The slope of decline was much steeper in the case of both MUNIX and muscle strength as examined by HHD. The MUNIX and HHD showed a marked decline even when the values of ALSFRS R and MRC were still stable ( P1, P2, and P3—resp. patient 1, patient 2, and patient 3).

ALSFRS R and MRC shared a similar deterioration pattern reaching down to 50 and 40% (resp.) of their initial value within 21 months of observation. The slope of decline was much steeper in the case of both MUNIX and muscle strength as examined by HHD (Figure 5). So was the range of decrease exceeding 90% in the observation period. There was a positive correlation between MUNIX and HHD throughout the study in merged results of all our patients (*r* = 0.8856, *p* < 0.0001). The MUNIX and HHD showed a marked decline even when the values of ALSFRS R and MRC were still stable. At the time of transplantation (from 3 to 6 months after the inclusion to the study), the ALSFRS R was 83%, 79%, 91%, and 68% of the value obtained at visit 1 (V1), whereas total MUNIX was as low as 68%, 85%, 59%, and 52% in patients P1, P2, P3, and P4, respectively (Figure 5). The above pattern was also present when upper and lower limbs were analyzed separately.

The values of three functional tests, which separately assessed the upper limbs, lower limbs, and the trunk, decreased faster and earlier than ALSFRS R. In patient P1, they all reached 50% of their initial value prior to V4, in patient P2 and P3 at V5 (except for TCT in P3), whereas ALSFRS R reached the same level at V5 (P1) and V7 (P3), or did not reach it yet (P2). Patient P4 only had 3 visits to date; his results could not be analyzed in this aspect.

**Figure 6:**
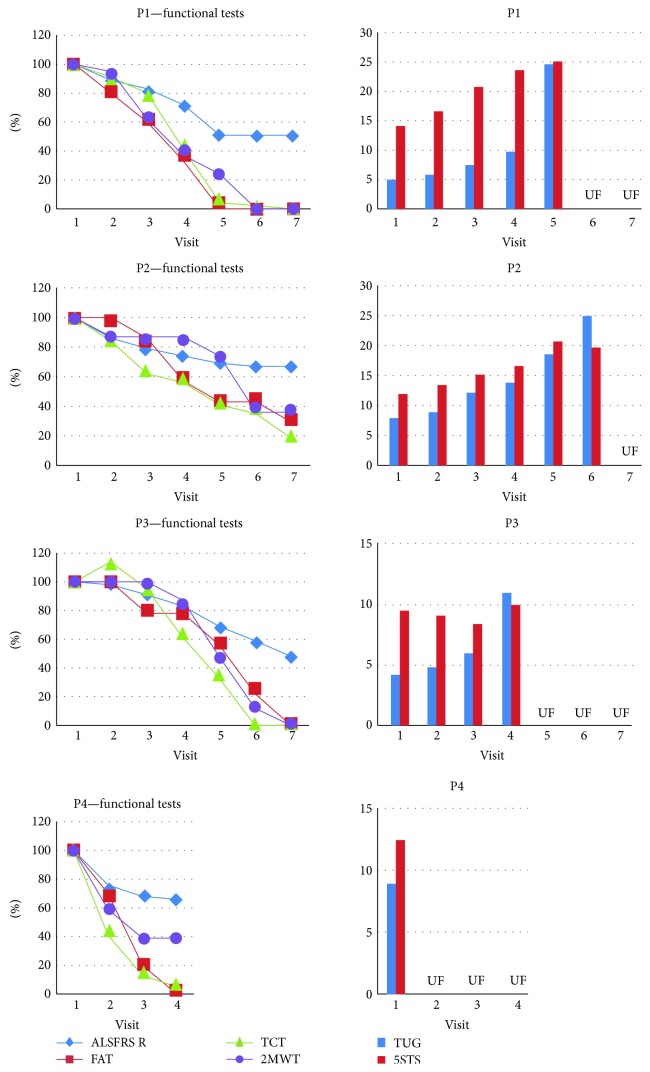
Comparison of the course of the disease before and after the cell therapy. The graphs show the course of disease preceding the use of ADRC (blue graph) and the course of disease during cell therapy.

The results of 2MWT matched those of MUNIX and HHD, but not MRC in the same extremities (Figures 4–6). The reduction of MUNIX values was an earlier event; however, the magnitude of 2MWT drop was higher. To a lesser extent, a similar correlation was found between the results of FAT, the only functional test assessing exclusively upper limbs, and MUNIX/HHD in the upper extremities (Figures 4–6).

Although 5STS and TUG showed good correlation with other functional parameters, their longitudinal use was limited in patients who were no longer able to stand up without the help of their arms/hands (on average 6–9 months after the first transplantation) (Figure 6).

The ALS patients differed in terms of spasticity, which—in all cases—increased with disease progression. The MAS results ranged from 1.06 (1.37 ± 0.25 in patient P1, 1.5 in P2, 0.0 in P3 and 1.37 ± 0.35 in P4) at the first visit to 1.84 (2.25 ± 0.7, 2.12 ± 1.06, 1 ± 1.4 and 2 ± 0.35, respectively) at the last follow-up visit (Vt). Due to spasticity, the MRC results could not be interpreted in P2 and P4. The spasticity did not correlate with fatigue (data not shown).

No differences in the progression rate of ALSFRS R was found between the pre- and posttransplantation observation period (Figure 5). There was a prominent increase in MUNIX results in P2 prior to intraspinal transplantation and 3 months after and in the posttransplantation period in the case of P4 (Figure 5). After intraspinal transplantation, MUNIX showed a higher progression rate in P1 and no changes in P3. The 2MWT decrease was stable in P1 and P3, whereas it was markedly increased in P2 and P4.

The FSS was found increased in all patients. It scored from the level of 39–50 at V1 to 50–68 at Vt.

The results of psychological assessment (ADI-2) showed mild depression (score 27) in two out of four patients prior to transplantation. It increased within the first 12 months of observation reaching the level of 30–33 (clinically relevant depression) in all patients (4/4) and required antidepressive treatment. None of the patients presented a wish for hastened death throughout the study. The QoL (ACSA) was very low in patients P2 and P4 (−5) and neutral in P1 (−1) and P3 (1). After 12 months, it markedly increased in the first two patients (1) and slightly decreased in P1 (−2) and P3 (−1).

### 3.2. Cell Culture: ADRC Parameters

Using a 1 ml suspension, we obtained approx. 14 × 10^6^/ml ADRC with 95% viability, expressing CD73 (99.3% of cells), CD90 (99.6%), and CD105 (89.1%) as well as CD34, CD19, CD11b, and HLA-DR (all in 1.7% of cells) as surface markers which was in line with our previous experiments (10). According to ISCT recommendation, the ability of the MSC population to create CFU (colony-forming unit fibroblasts) was considered one of the most specific tests to define the content of stem/progenitor cells in the whole heterogeneous MSC population, thus predicting expansion and longevity of the ADRC cells. Our results indicate that ADRCs isolated with the described method do contain a relatively high fraction of the genuine stem cells to approx. 20% of total (Figure 2(c)).

In the next task, the factors involved in self-renewal, regeneration, and immunomodulatory capacity of ADRC were analyzed in all CSF samples gathered according to the scheme given in Material and Methods. We have performed analysis of 102 cytokines/chemokines in the CSF of ALS patients in the samples taken before cell transplantation and then at 24 hours after each subsequent cell application, in order to correlate the change in their expression with course of the disease. Unfortunately, the pattern of their expression changed individually without any common trend neither for the whole group of patients nor for the particular stages of the disease (Figure 2(a)). Among these cytokines, only six factors have been identified, the levels of which would be correlated with the therapeutic effect of ADRC treatment. They exhibited a decrease in CSF levels of proinflammatory TNF*α*, IFN-*γ*, and IL-1*β* after MSC transplantation, but more interestingly with the exception of those taken 24 hours after the first ADRC injection when they remain unchanged or even elevated in comparison with the CSF samples from untreated patients. In contrast to this, growth factors and anti-inflammatory/regressive agents like IL-6, bFGF, and MMP-9 have been increased in most samples as well as those gathered at 24 hours after cell administration (Figure 2(b)).

## 4. Discussion

Qualification of ALS patients to controlled pharmacological clinical trials encounters a number of obstacles resulting from the disease character. They include prolonged diagnosis delay, variable clinical certainty at diagnosis, clinical phenotype conditioning disease progression, and a short ventilation-free survival [35]. Clinical trials including neurosurgical procedures, like in the case of intraspinal stem cell transplantation, additionally face the problem of bulbar and respiratory involvement interfering with anesthesia. In order to enable functional analysis, they also require a partially preserved function of muscles innervated by the studied part of the spinal cord. For these reasons, despite a high number of patients followed in our ALS center, their final eligibility for the current trial was only 3.2%. Our finding goes in line with recently published results from the French ALS registry where nearly 40% of patients had diagnosis delay > 12 months, over 30% of patients had a bulbar disease onset, 15% had other phenotype than classic ALS, and in nearly 30% of cases the diagnosis was only clinically possible [36]. When adding age limit, functional stage according to ALSFRS R, and the reduced FVC, the number of patients potentially eligible for a stem cell clinical trial dropped dramatically.

Intraspinal ADRC transplantation at the Th10/11 level was proved safe and well tolerated. In two patients, neurosurgery induced reduction of the muscle tone, which resulted in improved walking capacity of a one-month duration, objectively measured in one case. There was also a reduction in the progression rate of walking impairment within 3 months after the transplantation in the same patient. Both patients had a prevalent upper motor neuron involvement (UMN) with marked spasticity, and the effect was not observed after an intrathecal ADRC infusion. It suggests an intraspinal mechanism—either due to neurosurgical manipulation similar to postcontusion neurogenic shock or due to an immunomodulatory effect of the ADRC. Although the sedation effect could not be excluded within the first two days, both patients reported persistence of this state for the 4 following weeks. The phenomenon has not been previously reported following neurosurgical operations (if not after a pathologic mass removal). We did not observe any deterioration due to a reduced muscle tone in two other operated patients (including one with prevalent lower motor neuron (LMN) impairment) that could potentially decrease their physical performance.

Otherwise, the study did not prove effective in terms of reversing or relenting the disease progression. The limitations of the current study included an open-label character and a low patient number limiting the use of statistical analyses. However, a number of observations have been made. From all applied measures, MUNIX proved to be the first and the most sensitive tool in identifying fine changes at the muscle level. It was markedly more sensitive than ALSFRS R and MRC which was earlier found in electrophysiological studies in ALS [37]. Previous studies have also shown that unlike the majority of other ALS outcome measures (FVC, ALS-FRS, and survival), MUNIX has not been subject to high variability [38]. It also showed a high interrater reproducibility in patients with ALS [39]. Moreover, in longitudinal studies, it was reduced not only in paretic but also in presymptomatic muscles [40]. This feature is of utmost importance as it enables monitoring of fine changes during the stem cell therapies. The transplantations should however be planned earlier in the disease course since, as shown by the same group, at the time of diagnosis the total MUNIX score in ALS patients is reduced by 70% compared to healthy volunteers [41]. In an advanced process, the protective stem cell properties might not be sufficient to influence the clinical outcome.

Interestingly, in our hands MUNIX was higher in patients with prevalent UMN involvement. It may be due to the muscle fiber overwork accompanying spasticity. A similar phenomenon was described in Parkinson's disease. The authors found changed muscle's mechanical properties including increased muscle bulk related to structural changes within muscles accompanying pathological central neural drive [42]. Longitudinal studies on MUNIX in patients with marked UMN involvement as compared to those with preferential LMN damage should shed more light on this process. By that time, it is important to measure spasticity level in all patients who undergo intraspinal stem cell therapy. It might also be worth considering the use of averaging multiple MUNIX measures recently described in longitudinal assessment of ALS patients in order to further objectify results [43].

In our hand, the Modified Ashworth Scale was a sensitive tool in a potential patient stratification according to the spasticity level. A recent meta-analysis of studies regarding reliability of the scale showed a satisfactory inter- and intrarater agreement. MAS scores exhibited better reliability when measuring upper extremities compared to lower extremities [44]. It might explain the lack of score change in patient P4 who reported decreased muscle spasticity following the intraspinal transplantation procedure resulting in objective walking improvement.

In our current study, the dynamometry was the closest measurement to MUNIX, both on the upper and lower limb level. It was a fine tool in terms of slope and range of change in the course of the disease. The utility of this measure was proved in several other studies, showing a high reliability especially in early disease stages [19, 22, 42]. To our knowledge, to date it was not compared to MUNIX in longitudinal monitoring of the disease course.

Beside muscle and motor unit assessment, we found the functional tests very useful in a complex analysis of the preserved physical power of the limbs and trunk. ALSFRS R is the reference functional scale for all the clinical trials; it is simple and easy to use at every check-up visit. However, it asymmetrically addresses the functions of the bulbar muscles (12 points), upper (12 points), lower limbs (8 points), and trunk (4 points), together with respiratory functions (12 points). For this reason, an analysis of FAT, 2MWT, and TCT at the same time points allows for a more precise description of disease progression. By addressing the functions of one part of musculature at a time, this non-time-consuming examination (lasting up to 20 min altogether) gives a wider range of scores able to reflect more subtle changes over the disease course and applied treatment.

Although very useful in several neurologic conditions [45] as well as in elderly patients [30], 5STS and TUG have been found of limited use in monitoring the disease progression in patients with ALS. Since both techniques require standing up from a sitting position, patients with increased proximal muscles paresis were not able to perform the tests as early as 3 months after transplantation. Despite assessing trunk muscles in a more limited way, 2MTW was proved more easily applicable and showed a very early decrease with a wide range of change. Its combination with assessment of fatigability by RPE has given additional reassuring information (as p.ex. in P2 whose walking distance improved after the intraspinal transplantation which was accompanied by a marked decrease in fatigability by REP). Fatigue was reported by 83% of ALS patients [46]. It was the most prevalent symptom in the late stage of ALS, significantly decreasing the quality of life [47, 48]. It was also an important reason to give up physical activity [49]. In our study, all patients presented increased fatigue from the first visit. Longitudinal analysis showed its further increase with time. Despite close monitoring, multidisciplinary care, and participation in a clinical trial, all patients developed clinically relevant depression requiring pharmacological treatment. Since in the general ALS population, depression does not significantly increase with disease progression [50], the higher depression levels observed at the end of the study might have been due to the lack of improvement following the intervention. Interestingly, QoL did not go along with depression in individual cases. Primarily very low in 2 patients and neutral in the remaining two, it became neutral in all cases. The increase of QoL despite an apparent increase in motor impairment is probably due to the previously described adaptation process [51].

Beside a broad experience in patients' monitoring, there is still a need to find a correlation between the clinical management and quality of the used transplantation material. Analysis of MSC secretive properties in vitro and in vivo suggests the need for repeated/cyclic ADRC transplantation. It seems that the optimal time between consecutive transplantations in order to support their therapeutic effect is about 3 months [52]. This is in line with the first paper published in 2010 by our group, describing intracerebroventricular transplantation of cord blood-derived neural progenitors in a child with severe global brain ischemic injury. After a subsequent transplantation of the cells tagged with SPIO nanoparticles, we found them to have persisted at a wall of the lateral ventricle for about 4 months [53]. The levels of cytokines found in CSF at 3 months after ADRC transplantation are also in agreement with the data reported by others [54], stating that the route of intraspinal cell delivery is effective, feasible, and most probably optimal because of their strait vicinity or even incorporation into the host's neural network [55].

The cytokine and chemokine changes observed here after ADRC transplantation correspond well with the data published by Tyndall et al. [12]. Although abnormal levels of interleukin IL-6 are described mainly in relation to inflammatory processes [56], it should also be remembered that in reverse, IL-6 as belonging to the so-called dual-function cytokines may also inhibit TNF-alpha and IL-1*β* expression. Lu et al. [57] demonstrated in a cohort of ALS patients that IL-6 had a significantly increased expression at the end-stage of the disease. This may explain the increase found in our first patient (P1), the only one who has died during the course of observation. Conversely, in the other patients the level of IL-6 remained rather stable together with concomitant decrease of TNF-alpha and IL-1*β*. In light of the previously reported negative correlation between the levels of these cytokines and the duration and severity of ALS [58], this effect of ADRC which inhibits TNF-alpha and IL-1*β* expression seems to be therapeutically promising.

The above conclusion is also in line with other experiences gathered during preclinical experiments. They suggest that highly proliferating, undifferentiated, SRTF-expressing cells present in ADRC population are one of the most effective material in therapeutic transplantation.

To sum up, there is a strong need for an international collaborative effort facilitating an early and effective enrollment of ALS patients into stem cell clinical trials. It will help also to improve enrollment and monitoring criteria, which may result in more coherent and reliable data [59, 60].

Keeping in mind the limitations of the study, suggestions from the presented research indicate the following:

1. Enlargement of the group of patients at the early stage of disease selected from several centers
2. MUNIX analysis in patients with marked UMN involvement/together with MAS
3. Combination of MUNIX, HHD, MAS, 2MWT (with BS), FSS, FAT, and TCT (approx. 1 h) which may help obtain results along the disease progression

## Figures and Tables

**Table 1:** Inclusion and exclusion criteria for the intraspinal and intrathecal ADRC transplantation.

Inclusion criteria	Exclusion criteria
(i) Clinically definite or probable ALS	(i) PEG or indications for PEG insertion
(ii) Age 18–65 years	(ii) Respiratory insufficiency, NIPPV, IV, or indications
(iii) ALS − FRS > 25	(iii) *SOD1* and *C9orf72* mutation
(iv) FVC > 70%	(iv) Concomitant disorders with contraindications for neurosurgery
(v) Stable dose of riluzole for the past 30 days	(v) Pregnancy or lactation
(vi) Disease duration 6–24 months	(vi) Unable to provide informed consent
(vii) Pretreatment observation period of >3 months (neurological and physiotherapist assessment, ALSFRS R, and MUNIX)	
(viii) In females with childbearing potential at least two efficient contraceptive methods	
(ix) Able to provide informed consent	

**Table 2:** Timetable of the adipose-derived regenerative cell (ADRC) isolation and transplantation study.

Action visit	V1 screening	V2	V3	V4	V5	V6	V7
Week	−24	−12	0	12	24	36	48
Permitted delta in weeks to V1		+2		+2	+2	+/−2	+/−2
*Interventions*							
Intraspinal transplantation			X				
Intrathecal transplantation				X	X		
*Screening assessment*							
Patient information, informed consent	X		X	X	X		
Inclusion/exclusion criteria	X						
Medical history	X		X	X	X	X	X
Diagnosis according to EEC	X		X	X	X	X	X
Physical and neurological examination	X	X	X	X	X	X	X
EMG	X						
MUNIX	X	X	X	X	X	X	X
Spinal MRI C with contrast			X				
Spinal MRI Th with contrast			X	X	X		X
Spinal MRI S with contrast			X	X	X		X
Spinal MRI Th			X^∗^	X^∗^	X^∗^		
Spinal MRI LS			X^∗^	X^∗^	X^∗^		
Spirometry	X	X	X	X	X	X	X
Chest X-ray			X				
Laboratory studies	X		X	X	X		
*Muscle strength assessment*							
MRC	X	X	X	X	X	X	X
HHD	X	X	X	X	X	X	X
*Functional assessment*							
ALSFRS R	X	X	X	X	X	X	X
2MWT (with concomitant BS)	X	X	X	X	X	X	X
5STS	X	X	X	X	X	X	X
TUG	X	X	X	X	X	X	X
TCT	X	X	X	X	X	X	X
FAT	X	X	X	X	X	X	X
*Other examinations*							
FSS	X	X	X	X	X	X	X
MAS	X	X	X	X	X	X	X
*Psychological assessment*							
ADI-12	X		X		X		X
ACSA	X		X		X		X

MUNIX: motor unit number index (MUNIX). ^∗^Examination performer 48 h after the ARDC transplantation. MRC: Manual Muscle Test Medical Research Council; HHD: handheld dynamometer (HHD); ALSFRS R: amyotrophic lateral sclerosis functional rating scale-revised; 2MWT: two-minute walk test; BS: Borg scale; 5STS: Five Times Sit to Stand test; TUG: Timed Up and Go; FAT: Frenchay Arm Test; TCT: Trunk Control Test; MAS: Modified Ashworth Scale; FSS: Fatigue Severity Scale; ADI-12: ALS Depression Inventory; ACSA: Anamnestic Comparative Self-Assessment.

**Table 3:** Cytokine array assay.

Adiponectin/Acrp30	INF-gamma	Lipocalin-2/NGAL
Aggrecan	IGFBP-2	CCL2/MCP-1
Angiogenin	IGFBP-3	CCL7/MCP-3
Angiopoietin-1	IL-1 alpha/IL-1F1	M-CSF
Angiopoietin-2	IL-1 beta/IL-1F2	MIF
BAFF/BLyS/TNFSF13B	IL-1ra/IL-1F3	CXCL9/MIG
BDNF	IL-2	CCL3/CCL4 MIP-1 alpha/beta
CD14	IL-3	CCL20/MIP-3 alpha
CD30	IL-4	CCL19/MIP-3 beta
CD40 ligand/TNFSF5	IL-5	MMP-9
Chitinase 3-like	IL-6	Myeloperoxidase
Complement component C5/C5a	IL-8	Osteopontin (OPN)
Complement factor D	IL-10	PDGF-AA
C-reactive protein (CRP)	IL-11	PDGF-AB/BB
Cripto-1	IL-12 p70	Pentraxin 3/TSF-14
Cystatin C	IL-13	CXCL4/PF4
Dkk-1	IL-15	RAGE
DPPIV/CD26	IL-16	CCL5/RANTES
EGF	IL-17A	RBP4
CXCL5/ENA-78	IL-18 BPa	Relaxin-2
Endoglin/CD105	IL-19	Resistin
EMMPRIN	IL-22	CXCL12/SDF-1 alpha
Fas ligand	IL-23	Serpin E1/PAI-1
FGF basic	IL-24	SHBG
KGF/FGF-7	IL-27	ST2/IL1 R4
FGF-19	IL-31	CCL17/TARC
Fit-3 ligand	IL-32alpha/beta/gamma	TFF3
G-CSF	IL-33	TfR
GDF-15	IL-34	TGF-alpha
GM-CSF	CXCL10/IP-10	Thrombospondin-1
CXCL1/GRO alpha	CXCL11/I-TAC	TNF-alpha
Growth hormone (GH)	Kallikrein 3/PSA	uPAR
HGF	Leptin	VEGF
ICAM-1/CD54	LIF	Vitamin D BP

**Table 4:** Longitudinal forced vital capacity assessment in ALS patients prior to and after ADRC administration.

	Patient/visit	V1	V2	V3 (prior to intraspinal tx)	V4 (prior to 1st intrathecal tx)	V5 (prior to 2nd intrathecal tx)	V6	V7
FVC (%)	P1	110	105	100	100	90	85	80
	P2	89	89	84	88	85	75	65 (NIV)
	P3	128	128	106	106	100	90	83
	P4		90	71	65	44		

## Data Availability

The data from clinical study used to support the findings of this study are available from Professor Magdalena Kuzma-Kozakiewicz (mkuzma@wum.edu.pl) and from a basic study from the corresponding author upon request.
